# Invasive Cancer Incidence, 2004–2013, and Deaths, 2006–2015, in Nonmetropolitan and Metropolitan Counties — United States

**DOI:** 10.15585/mmwr.ss6614a1

**Published:** 2017-07-07

**Authors:** S. Jane Henley, Robert N. Anderson, Cheryll C. Thomas, Greta M. Massetti, Brandy Peaker, Lisa C. Richardson

**Affiliations:** 1 National Center for Chronic Disease Prevention and Health Promotion, CDC; 2 National Center for Health Statistics, CDC

## Abstract

**Problem/Condition:**

Previous reports have shown that persons living in nonmetropolitan (rural or urban) areas in the United States have higher death rates from all cancers combined than persons living in metropolitan areas. Disparities might vary by cancer type and between occurrence and death from the disease. This report provides a comprehensive assessment of cancer incidence and deaths by cancer type in nonmetropolitan and metropolitan counties.

**Reporting Period:**

2004–2015.

**Description of System:**

Cancer incidence data from CDC’s National Program of Cancer Registries and the National Cancer Institute’s Surveillance, Epidemiology, and End Results program were used to calculate average annual age-adjusted incidence rates for 2009–2013 and trends in annual age-adjusted incidence rates for 2004–2013. Cancer mortality data from the National Vital Statistics System were used to calculate average annual age-adjusted death rates for 2011–2015 and trends in annual age-adjusted death rates for 2006–2015. For 5-year average annual rates, counties were classified into four categories (nonmetropolitan rural, nonmetropolitan urban, metropolitan with population <1 million, and metropolitan with population ≥1 million). For the trend analysis, which used annual rates, these categories were combined into two categories (nonmetropolitan and metropolitan). Rates by county classification were examined by sex, age, race/ethnicity, U.S. census region, and cancer site. Trends in rates were examined by county classification and cancer site.

**Results:**

During the most recent 5-year period for which data were available, nonmetropolitan rural areas had lower average annual age-adjusted cancer incidence rates for all anatomic cancer sites combined but higher death rates than metropolitan areas. During 2006–2015, the annual age-adjusted death rates for all cancer sites combined decreased at a slower pace in nonmetropolitan areas (-1.0% per year) than in metropolitan areas (-1.6% per year), increasing the differences in these rates. In contrast, annual age-adjusted incidence rates for all cancer sites combined decreased approximately 1% per year during 2004–2013 both in nonmetropolitan and metropolitan counties.

**Interpretation:**

This report provides the first comprehensive description of cancer incidence and mortality in nonmetropolitan and metropolitan counties in the United States. Nonmetropolitan rural counties had higher incidence of and deaths from several cancers related to tobacco use and cancers that can be prevented by screening. Differences between nonmetropolitan and metropolitan counties in cancer incidence might reflect differences in risk factors such as cigarette smoking, obesity, and physical inactivity, whereas differences in cancer death rates might reflect disparities in access to health care and timely diagnosis and treatment.

**Public Health Action:**

Many cancer cases and deaths could be prevented, and public health programs can use evidence-based strategies from the U.S. Preventive Services Task Force and Advisory Committee for Immunization Practices (ACIP) to support cancer prevention and control. The U.S. Preventive Services Task Force recommends population-based screening for colorectal, female breast, and cervical cancers among adults at average risk for these cancers and for lung cancer among adults at high risk; screening adults for tobacco use and excessive alcohol use, offering counseling and interventions as needed; and using low-dose aspirin to prevent colorectal cancer among adults considered to be at high risk for cardiovascular disease based on specific criteria. ACIP recommends vaccination against cancer-related infectious diseases including human papillomavirus and hepatitis B virus. *The Guide to Community Preventive Services* describes program and policy interventions proven to increase cancer screening and vaccination rates and to prevent tobacco use, excessive alcohol use, obesity, and physical inactivity.

## Introduction

Cancer rates vary by many different characteristics, including geographic classifications such as county of residence. County populations differ from one another on the basis of demographic, environmental, economic, and social characteristics, which might influence the magnitude and types of health problems, including cancer (*1*). Counties can be classified as metropolitan or nonmetropolitan as defined by the Office of Management and Budget (*2*). This classification scheme can be further categorized by using the U.S. Department of Agriculture Economic Research Service (USDA ERS) rural-urban continuum codes classification scheme, which categorizes metropolitan counties by the population size of their metropolitan area and nonmetropolitan counties by degree of urbanization and adjacency to a metropolitan area (*3*). Previous research indicates that although cancer death rates decreased from 1999 to 2014 among all counties, decreases were slower in nonmetropolitan areas than metropolitan areas, increasing disparities (*1*). Comparisons of cancer death rates in nonmetropolitan areas and metropolitan areas have been described previously but not systematically assessed (*1*,*4*).

The *Healthy People 2020* initiative established a set of goals and objectives designed to improve the health of the U.S. population, recognizing the important role played by determinants of health, such as geographic location (*5*). As of 2017, *Healthy People 2020* includes objectives for reducing age-adjusted overall cancer death rates to 161.4 deaths per 100,000 persons, lung cancer to 45.5 deaths per 100,000 persons, female breast cancer to 20.7 deaths per 100,000 females, cervical cancer to 2.2 deaths per 100,000 females, colorectal cancer to 14.5 deaths per 100,000 persons, oral cavity and pharynx cancer to 2.3 deaths per 100,000 persons, prostate cancer to 21.8 deaths per 100,000 males, and melanoma to 2.4 deaths per 100,000 persons (*5*). *Healthy People 2020* also includes objectives to reduce age-adjusted incidence rates of colorectal cancer to 39.9 cases per 100,000 persons, late-stage breast cancer to 42.2 cases per 100,000 females, and cervical cancer to 7.3 cases per 100,000 females (*5*).

This report assesses differences in cancer incidence and death rates by county classification, age group, sex, race/ethnicity, U.S. census region, and cancer type (and stage for incidence of cancers that are detectable by screening), as well as changes over time in incidence and death rates for common cancers in nonmetropolitan and metropolitan counties and whether these trends differ. In addition, this report assesses progress toward reaching the *Healthy People 2020* objectives for cancer (*5*). These findings can be used by public health officials to identify areas with the highest rates of cancer incidence or mortality and focus on regions that might benefit from strategies to prevent cancer from occurring or to reduce deaths from cancer. 

## Methods

To examine differences in cancer incidence and mortality in nonmetropolitan and metropolitan counties in the United States, CDC analyzed cancer incidence data from CDC’s National Program of Cancer Registries (NPCR) and the National Cancer Institute’s (NCI’s) Surveillance, Epidemiology, and End Results (SEER) program and cancer mortality data from the National Vital Statistics System (NVSS) (*6*,*7*). The most recent data available are through 2013 for incidence and through 2015 for deaths. This report presents average annual age-adjusted rates for the most recent 5-year period with available data (2009–2013 for incidence and 2011–2015 for deaths) and trends in annual age-adjusted incidence rates for the most recent 10-year period with available data (2004–2013 for incidence and 2006–2015 for deaths). For 5-year average annual rates, counties were classified into four categories (nonmetropolitan rural, nonmetropolitan urban, metropolitan with population <1 million, and metropolitan with population ≥1 million). For the trend analysis, which used annual rates, these categories were combined into two categories (nonmetropolitan and metropolitan).

### Incidence Data

Data on new cases of cancer diagnosed during 2004–2013 were obtained from population-based cancer registries affiliated with NPCR and SEER programs in each state and the District of Columbia. Data from Nevada did not meet U.S. Cancer Statistics publication criteria for 2004–2013, and county-level data were not available for Kansas and Minnesota; consequently, incidence data in this report represent 97% of the U.S. population. Cases were classified first by anatomic site (https://seer.cancer.gov/siterecode), and cases with hematopoietic histologies were classified further (*7*). Only cases of invasive cancer were included, except for urinary bladder cancer, for which in situ tumors also were included. Cancers for which population-based screening recommendations exist (breast, cervix, colon and rectum, and lung) also were characterized by stage at diagnosis; SEER Summary Stage 2000 was used to characterize cancers as localized, regional, distant, or unknown stage using clinical and pathologic tumor characteristics, such as tumor size, depth of invasion and extension to regional or distant tissues, involvement of regional lymph nodes, and distant metastases (https://seer.cancer.gov/tools/ssm). Late-stage cancers included those diagnosed at the regional or distant stage.

### Mortality Data

Data on cancer deaths were based on death certificates registered during 2006–2015 by the 50 states and the District of Columbia and compiled into a national file through NVSS. The underlying cause of death was based on the *International Classification of Diseases, 10th Revision* (ICD-10) codes (*8*) and categorized according to SEER cancer site groups to maximize comparability with the *International Classification of Diseases* *for Oncology, third edition* (ICD-O-3) classification (https://seer.cancer.gov/codrecode). Mortality data in this report cover 100% of the U.S. population.

### County Classification

The USDA ERS rural-urban continuum codes (2013 vintage) were used to categorize county residence at time of cancer diagnosis or death as nonmetropolitan or metropolitan; nonmetropolitan counties were further categorized by degree of urbanization and adjacency to a metropolitan area and metropolitan counties by the population size of their metropolitan area (https://www.ers.usda.gov/data-products/rural-urban-continuum-codes). Thus, counties were more specifically classified as nonmetropolitan rural (rural-urban continuum codes 8 and 9) or nonmetropolitan urban (rural-urban continuum codes 4–7), and metropolitan counties were classified as metropolitan with <1 million population (rural-urban continuum codes 2 and 3) or metropolitan with ≥1 million population (rural-urban continuum code 1). The two-category classification was used for the trend analysis, which used annual rates; the rate analysis, which used 5-year average annual rates, allowed for the more detailed four-category classification.

### Demographic and Tumor Characteristics

Information about race/ethnicity was collected separately and was based on medical records or death certificates. In this report, information about race/ethnicity was combined to create five mutually exclusive groups: non-Hispanic white, non-Hispanic black, non-Hispanic American Indian/Alaska Native (AI/AN), non-Hispanic Asian/Pacific Islander (API), and Hispanic. Cases among persons with other or unknown race (2%) or unknown ethnicity (2%) were included in overall rates but were not included as separate categories.

Individual cancer sites included the most common cancers among men and among women in each of the five racial/ethnic groups. These sites include prostate; female breast; lung and bronchus (lung); colon and rectum (colorectal); oral cavity and pharynx; esophagus; stomach; liver and intrahepatic bile duct (liver); pancreas; larynx; corpus uteri and uterus, not otherwise specified (uterus); uterine cervix (cervix); ovary; kidney and renal pelvis (kidney); urinary bladder (bladder); thyroid; melanoma; brain and other central nervous system (brain); non-Hodgkin lymphoma; myeloma; and leukemia.

### Statistical Analysis

Population estimates for rate denominators were a modification of annual county population estimates by age, sex, bridged race, and ethnicity produced by the U.S. Census Bureau in collaboration with CDC and with support from NCI (https://seer.cancer.gov/popdata). The 5-year average annual incidence rates for 2009–2013 and 5-year average annual death rates for 2011–2015 per 100,000 persons were age-adjusted to the 2000 U.S. standard population. Rates were estimated by nonmetropolitan (urban and rural) and metropolitan residence (population size <1 million and ≥1 million), sex, age group (<20, 20–44, 45–64, 65–74, and ≥75 years), racial/ethnic group, U.S. census region, and cancer site. Rate ratios were calculated to compare incidence and death rates in nonmetropolitan rural counties with rates in other counties; rates were considered significantly different (p<0.05) if the 95% confidence interval (CI) for the rate ratio excluded one. The average annual percentage change (AAPC) was used to quantify changes in annual age-adjusted incidence rates during 2004–2013 and annual age-adjusted death rates during 2006–2015 and was calculated using joinpoint regression, which allowed different slopes for two periods; the year at which slopes changed could vary by county classification (https://surveillance.cancer.gov/joinpoint). To determine whether AAPC was significantly different from zero, a t-test was used for 0 joinpoints, and a z-test was used for 1 joinpoint. Rates were considered to increase if AAPC >0 (p<0.05) and to decrease if AAPC <0 (p<0.05); otherwise rates were considered stable. Differences in AAPCs between nonmetropolitan and metropolitan counties were considered significant (p<0.05) if the 95% CI for the difference excluded zero. All statistical tests were two sided. 

## Results

### Cancer Incidence Rates and Trends

Cancer cases in nonmetropolitan rural counties comprised 2% of all cancers in this analysis, cancer cases in nonmetropolitan urban counties comprised 15%, and cases in all metropolitan counties accounted for 83% (Table 1). The average annual age-adjusted incidence rate (incidence rate) for all anatomic cancer sites combined was lower among nonmetropolitan rural counties (442 cases per 100,000 persons) than rates among nonmetropolitan urban counties (455 cases per 100,000 persons), metropolitan counties with <1 million population (456 cases per 100,000 persons), and metropolitan counties with ≥1 million population (457 cases per 100,000 persons). Overall cancer incidence rates among men and among women were lower among nonmetropolitan rural counties than in other counties. By age, overall cancer incidence rates among nonmetropolitan rural counties were similar to those in other counties among those aged <45 years but lower among those aged ≥45 years. Among non-Hispanic whites, non-Hispanic blacks, and Hispanics, overall cancer incidence rates were lower in nonmetropolitan rural counties than in other counties; however, among AI/ANs, the highest rates were in nonmetropolitan rural counties. Overall cancer incidence rates among APIs did not differ by county classification.

**TABLE 1:** Average annual number and average annual age-adjusted rate*of invasive cancer cases in nonmetropolitan and metropolitan counties,^†^ by selected characteristics — United States,^§^ 2009–2013

Characteristic	Nonmetropolitan counties				Metropolitan counties			
	Rural		Urban		Population <1 million		Population ≥1 million	
	No.	Rate	No.	Rate	No.	Rate	No.	Rate
**All sites (total)**	**27,419**	**442.4**	**223,103**	**455.0^¶^**	**474,503**	**455.7^¶^**	**786,821**	**457.3^¶^**
**Sex**								
Male	14,959	496.6	118,289	512.7^¶^	244,277	510.6^¶^	393,408	511.3^¶^
Female	12,460	399.9	104,814	412.8^¶^	230,226	416.5^¶^	393,413	420.9^¶^
**Age group (yrs)**								
<20	191	17.8	1,772	17.3	4,372	17.6	8,116	18.4
20–44	1,352	114.8	13,095	116.8	32,680	117.2	63,176	115.8
45–64	9,636	665.5	80,782	680.2^¶^	175,389	678.4^¶^	303,060	672.1^¶^
65–74	8,350	1788.2	64,814	1855.2^¶^	129,874	1840.1^¶^	201,855	1845.7^¶^
≥75	7,890	2146.1	62,641	2218.3^¶^	132,187	2249.1^¶^	210,615	2297.4^¶^
Race/Ethnicity**								
White	22,907	444.0	192,001	459.4^¶^	384,155	464.5^¶^	540,902	480.4^¶^
Black	1,600	454.7	14,448	467.2^¶^	38,768	473.4^¶^	101,766	478.5^¶^
American Indian/Alaska Native	538	490.6	2,766	399.9^¶^	2,422	374.9^¶^	1,792	315.7^¶^
Asian/Pacific Islander	46	310.4	1,312	314.5	8,972	313.4	32,029	294.1
Hispanic	330	290.5	6,262	331.2^¶^	27,615	354.4^¶^	75,637	360.4^¶^
**Census region**								
Northeast	1,084	468.1	28,923	486.1^¶^	81,675	492.1^¶^	206,958	491.9^¶^
Midwest	9,338	442.2	66,757	456.4^¶^	94,999	460.2^¶^	139,844	475.9^¶^
South	14,412	448.6	97,684	457.8^¶^	202,444	453.7^¶^	258,686	446.1
West	2,586	402.4	29,740	418.3^¶^	95,385	429.3^¶^	181,333	425.8^¶^
**Cancer site**								
Female breast	3,337	108.3	28,584	114.0^¶^	66,806	122.2^¶^	117,971	126.5^¶^
Late stage	1,197	40.4	9,960	40.9	22,556	42.2^¶^	40,359	43.6^¶^
Lung and bronchus	4,589	70.6	35,420	70.0	67,973	64.5^¶^	99,652	59.1^¶^
Late stage	3,353	51.5	25,695	50.7	49,491	46.8^¶^	71,744	42.3^¶^
Colon and rectum	2,749	43.9	21,526	43.8	41,209	39.6^¶^	68,778	40.2^¶^
Late stage	1,508	24.3	11,741	24.0	22,255	21.5^¶^	37,248	21.8^¶^
Cervix	190	8.3	1,675	8.3	3,533	7.5^¶^	6,632	7.6^¶^
Late stage	100	4.0	864	4.1	1,770	3.6^¶^	3,257	3.6
Prostate	3,600	111.3	28,027	114.1^¶^	60,569	120.0^¶^	102,964	127.6^¶^
Oral cavity and pharynx	733	11.9	6,059	12.2	12,301	11.6	19,378	10.9^¶^
Esophagus	316	4.8	2,587	5.1	5,209	4.9	7,634	4.4^¶^
Stomach	343	5.4	2,847	5.8^¶^	6,368	6.1^¶^	12,454	7.3^¶^
Liver and intrahepatic bile duct	369	5.7	3,272	6.4^¶^	7,782	7.2^¶^	15,132	8.4^¶^
Pancreas	745	11.7	6,008	12.0	12,868	12.2^¶^	21,624	12.7^¶^
Larynx	285	4.4	2,200	4.3	3,979	3.7^¶^	5,739	3.2^¶^
Melanoma	1,149	19.9	9,298	20.0	22,118	21.9^¶^	32,841	19.2^¶^
Corpus uteri and uterus, not otherwise specified	757	23.7	6,537	25.2^¶^	14,159	24.9^¶^	25,215	26.2^¶^
Ovary	339	11.1	2,835	11.3	6,331	11.5	11,092	11.8^¶^
Urinary bladder	1,240	19.3	10,563	21.1^¶^	22,083	21.1^¶^	33,897	20.2^¶^
Kidney and renal pelvis	1,009	16.6	8,169	16.8	16,879	16.3	27,173	15.7^¶^
Brain and other nervous system	352	6.4	3,025	6.7	6,644	6.7	11,021	6.5
Thyroid	567	11.4	5,019	12.0^¶^	12,866	13.5^¶^	25,265	14.8^¶^
Myeloma	1,091	17.8	8,832	18.3	19,322	18.8^¶^	32,933	19.4^¶^
Non-Hodgkin lymphoma	368	5.7	2,974	5.9	6,497	6.2^¶^	11,397	6.7^¶^
Leukemia	786	13.3	6,456	13.7	13,644	13.5	22,474	13.4
Other cancers	2,505	42.9	21,190	45.1^¶^	45,361	44.7^¶^	75,557	44.8^¶^

* Per 100,000 persons, age-adjusted to the 2000 U.S. standard population.

^†^ Counties were identified using the U.S. Department of Agriculture Economic Research Service 2013 vintage rural-urban continuum code, which categorizes nonmetropolitan counties by degree of urbanization and adjacency to a metropolitan area, and metropolitan counties by the population size of their metropolitan area (https://www.ers.usda.gov/data-products/rural-urban-continuum-codes).

^§^ Cancer incidence data were compiled from cancer registries that meet the data quality criteria for all invasive cancer sites combined, representing approximately 97% of the U.S. population. (Data from Nevada did not meet U.S. Cancer Statistics publication criteria for 2004–2013, and county-level data were not available for Kansas and Minnesota.) Late-stage cancers include those diagnosed at the regional or distant stage, after the cancer has spread.

^¶^ Rates were significantly different from rates in nonmetropolitan rural areas. (The 95% confidence interval for the rate ratio excluded one.)

** Mutually exclusive racial/ethnic groups are based on information about race/ethnicity that was collected separately and combined for this report. The white, black, American Indian/Alaska Native, and Asian/Pacific Islander race categories are all non-Hispanic. Rates are not presented for those with unknown or other race or unknown ethnicity. Data from Virginia were excluded because the ethnicity for a large percentage was unknown.

**FIGURE 1:**
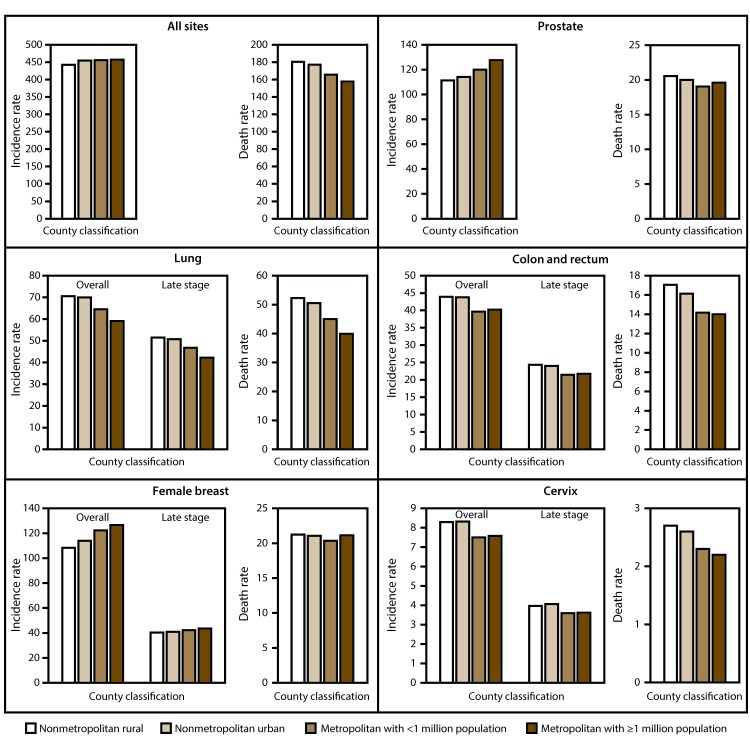
Average annual age-adjusted rates* of new cases^†^ of common cancers (2009–2013) and deaths^§^ from common cancers (2011–2015) in nonmetropolitan and metropolitan counties^¶^ — United States * Per 100,000 persons, age-adjusted to the 2000 U.S. standard population. ^†^ Cancer incidence data compiled from cancer registries that meet the data quality criteria for all invasive cancer sites combined, representing approximately 97% of the U.S. population. (Data from Nevada did not meet U.S. Cancer Statistics publication criteria for 2004–2013, and county-level data were not available for Kansas and Minnesota.) Late-stage cancers include those diagnosed at the regional or distant stage, after the cancer has spread. ^§^ Cancer mortality data are from the National Vital Statistics System (representing 100% of the U.S. population). ^¶^ Counties were identified using the U.S. Department of Agriculture Economic Research Service 2013 vintage rural-urban continuum code, which categorizes nonmetropolitan counties by degree of urbanization and adjacency to a metropolitan area, and metropolitan counties by the population size of their metropolitan area (https://www.ers.usda.gov/data-products/rural-urban-continuum-codes).

Although many differences in cancer site between nonmetropolitan rural and urban counties were not significant, nonmetropolitan rural counties generally experienced lower incidence rates than metropolitan counties for female breast cancer, late-stage female breast cancer, and prostate cancer but higher incidence rates for lung cancer, late-stage lung cancer, colorectal cancer, late-stage colorectal cancer, and cervical cancer (Figure 1). Nonmetropolitan rural counties experienced lower incidence rates than all other counties for cancers of the stomach, liver, uterus, bladder, and thyroid and lower incidence rates than metropolitan counties (but similar to nonmetropolitan urban counties) for pancreatic cancer, myeloma, non-Hodgkin lymphoma, and other cancers; higher incidence rates than metropolitan counties for laryngeal cancer; higher incidence rates than metropolitan counties with population ≥1 million population for cancers of the oral cavity and pharynx, esophagus, and kidney and for melanoma; and rates similar to those of other counties for brain cancer and leukemia (Table 1). 

**FIGURE 2:**
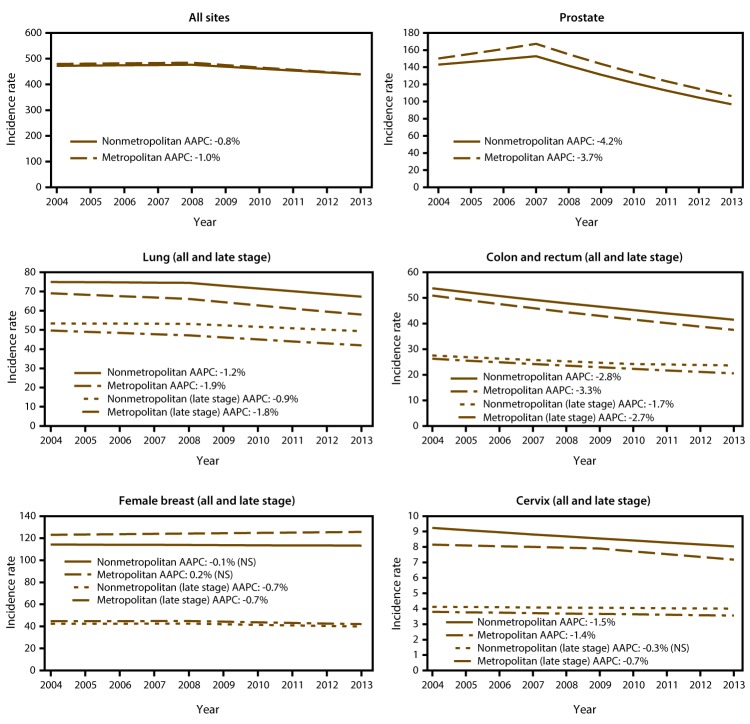
Trends* in annual age-adjusted incidence rates^†^ among persons of all ages for common cancers in nonmetropolitan and metropolitan counties,^§^ by year of diagnosis — United States, 2004–2013 **Abbreviations:** AAPC = average annual percentage change; NS = not significant. * Trends were measured with AAPC in annual rates (per 100,000, age-adjusted to the 2000 U.S. standard population) calculated using joinpoint regression, which allowed different slopes for two periods; the year at which slopes changed could vary by county classification. To determine whether AAPC was significantly different from zero, a t-test was used for 0 joinpoints, and a z-test was used for 1 joinpoint. Rates were considered to increase if AAPC >0 (p<0.05) and to decrease if AAPC <0 (p<0.05); otherwise rates were considered stable. All AAPCs were significantly different (p<0.05) from zero unless otherwise indicated (by NS). ^†^ Cancer incidence data were compiled from cancer registries that meet the data quality criteria for all invasive cancer sites combined, representing approximately 97% of the U.S. population. (Data from Nevada did not meet U.S. Cancer Statistics publication criteria for 2004–2013, and county-level data were not available for Kansas and Minnesota.) Late-stage cancers include those diagnosed at the regional or distant stage, after the cancer has spread. ^§^ Nonmetropolitan and metropolitan counties were identified using the U.S. Department of Agriculture Economic Research Service 2013 vintage rural-urban continuum code (https://www.ers.usda.gov/data-products/rural-urban-continuum-codes). AAPCs differed significantly between nonmetropolitan and metropolitan counties for cancers of the lung and colon and rectum but did not differ for cancers of all sites, female breast, prostate, or cervix.

During 2004–2013, overall cancer incidence rates decreased similarly in nonmetropolitan counties (-0.8% per year) and in metropolitan counties and (-1% per year) (Figure 2). Although overall breast cancer incidence rates were stable, late-stage breast cancer incidence rates decreased 0.7% per year both in nonmetropolitan and metropolitan counties (Figure 2). Incidence rates for cervical and prostate cancers decreased at similar rates in nonmetropolitan and metropolitan counties, whereas incidence rates for cancer of the colon and rectum (all and late-stage) and lung cancer (all and late-stage), which were already higher in nonmetropolitan counties, decreased more slowly than in metropolitan counties, increasing existing disparities (Figure 2). Some cancer incidence rates decreased at similar rates in nonmetropolitan and metropolitan counties (ovarian and brain cancers and non-Hodgkin lymphoma), whereas some decreased more slowly in nonmetropolitan counties (esophageal and laryngeal cancer) (supplemental Table 1 https://stacks.cdc.gov/view/cdc/45451). Incidence rates for myeloma, melanoma, and cancers of the pancreas, kidney, and thyroid increased at similar rates in nonmetropolitan and metropolitan counties; rates for cancers of the oral cavity and pharynx and liver increased more quickly in nonmetropolitan counties; and rates for uterine cancer increased more quickly in metropolitan counties (supplemental Table 1 https://stacks.cdc.gov/view/cdc/45451). Bladder cancer incidence rates increased 1.1% per year in metropolitan counties but decreased 0.5% per year in nonmetropolitan counties (supplemental Table 1 https://stacks.cdc.gov/view/cdc/45451).

### Cancer Death Rates and Trends

An average of 48,194 cancer deaths occurred each year during 2011–2015 in nonmetropolitan rural counties, representing 8% of all cancer deaths reported in the United States (Table 2). The average annual age-adjusted death rate (death rate) for all cancer sites combined was higher in nonmetropolitan rural counties (180 deaths per 100,000 persons) than in nonmetropolitan urban counties (177 deaths per 100,000 persons), metropolitan counties with <1 million population (166 deaths per 100,000 persons), and metropolitan counties with ≥1 million population (158 deaths per 100,000 persons). By sex, overall cancer death rates among both men and women were higher in nonmetropolitan rural counties than in other counties. By age, overall cancer death rates in nonmetropolitan rural counties were generally similar to those in other counties among those aged <20 years but higher among those aged ≥20 years. Overall cancer death rates were higher in nonmetropolitan rural counties than in other counties among non-Hispanic whites and AI/ANs but lower among Hispanics and APIs; among non-Hispanic blacks, rates in nonmetropolitan rural counties were lower than in nonmetropolitan urban counties but higher than in metropolitan counties.

**TABLE 2:** Average annual number and average annual age-adjusted rate*of cancer deaths in nonmetropolitan and metropolitan counties,^†^ by selected characteristics — United States,^§^ 2011–2015

Characteristic	Nonmetropolitan counties				Metropolitan counties			
	Rural		Urban		Population <1 million		Population ≥1 million	
	No.	Rate	No.	Rate	No.	Rate	No.	Rate
**All sites (total)**	**48,194**	**180.4**	**61,142**	**177.2^¶^**	**184,261**	**165.5^¶^**	**292,767**	**157.8^¶^**
**Sex**								
Male	26,741	218.3	33,214	214.5^¶^	98,208	199.6^¶^	149,952	187.9^¶^
Female	21,454	150.7	27,929	149.0^¶^	86,052	140.2^¶^	142,815	136.9^¶^
**Age group (yrs)**								
<20	103	2.2	156	2.2	569	2.3	1,077	2.4^¶^
20–44	1,021	19.0	1,402	16.6^¶^	4,752	15.2^¶^	8,702	14.3^¶^
45–64	12,810	237.0	17,314	226.5^¶^	49,944	202.7^¶^	80,818	176.3^¶^
65–74	13,741	689.7	16,998	678.2^¶^	49,374	622.7^¶^	75,031	590.8^¶^
≥75	20,518	1331.2	25,871	1334.3	79,619	1287.9^¶^	127,135	1289.3^¶^
Race/Ethnicity**								
White	42,955	180.9	54,281	178.8^¶^	154,391	168.4^¶^	212,144	163.5^¶^
Black	3,560	202.9	4,119	209.0^¶^	16,400	197.3^¶^	43,245	191.2^¶^
American Indian/Alaska Native	731	181.2	660	152.6^¶^	887	135.5^¶^	665	110.8^¶^
Asian/Pacific Islander	79	85.7	427	111.4^¶^	3,274	108.6^¶^	11,949	100.7^¶^
Hispanic	774	108.7	1,573	115.4^¶^	8,970	116.2^¶^	23,869	113.3^¶^
**Census region**								
Northeast	3,740	175.2	7,468	169.7^¶^	29,042	165.1^¶^	69,514	157.8^¶^
Midwest	16,352	175.0	20,326	177.2^¶^	38,898	171.4^¶^	60,419	170.2^¶^
South	23,739	190.9	24,503	188.0^¶^	80,333	170.4^¶^	95,175	159.1^¶^
West	4,364	153.7	8,845	157.4^¶^	35,988	150.9^¶^	67,659	146.4^¶^
**Cancer site**								
Female breast	2,939	21.2	3,879	21.1	12,299	20.3^¶^	22,020	21.1
Lung and bronchus	14,284	52.2	17,705	50.5^¶^	50,430	45.0^¶^	73,544	39.9^¶^
Colon and rectum	4,535	17.1	5,531	16.1^¶^	15,717	14.2^¶^	26,050	14.0^¶^
Cervix	310	2.7	396	2.6^¶^	1,221	2.3^¶^	2,208	2.2^¶^
Prostate	2,343	20.5	2,844	20.0^¶^	8,674	19.0^¶^	14,157	19.6^¶^
Oral cavity and pharynx	777	3.0	978	2.8^¶^	2,892	2.6^¶^	4,470	2.3^¶^
Esophagus	1,242	4.6	1,635	4.7	4,859	4.3^¶^	7,050	3.7^¶^
Stomach	717	2.8	923	2.7	3,159	2.9^¶^	6,427	3.5^¶^
Liver and intrahepatic bile duct	1,580	5.8	2,153	6.1^¶^	7,233	6.3^¶^	12,849	6.7^¶^
Pancreas	2,970	11.0	3,867	11.1	12,208	10.9	20,390	11.0
Larynx	332	1.2	418	1.2	1,173	1.0^¶^	1,806	1.0^¶^
Melanoma	759	3.0	984	3.0	3,082	2.8^¶^	4,372	2.4^¶^
Corpus uteri and uterus, not otherwise specified	615	4.3	841	4.4	2,701	4.4	5,184	4.9^¶^
Ovary	1,040	7.4	1,363	7.3	4,414	7.2	7,411	7.1^¶^
Urinary bladder	1,229	4.6	1,608	4.7	4,896	4.4^¶^	7,877	4.3^¶^
Kidney and renal pelvis	1,210	4.6	1,493	4.3^¶^	4,487	4.0^¶^	6,680	3.6^¶^
Brain and other nervous system	1,158	4.6	1,544	4.7	4,914	4.5	7,860	4.2^¶^
Thyroid	135	0.6	163	0.5	536	0.5^¶^	969	0.5
Myeloma	900	3.3	1,168	3.4	3,741	3.4	6,067	3.3
Non-Hodgkin lymphoma	1,583	6.0	2,118	6.2^¶^	6,410	5.8^¶^	10,162	5.6^¶^
Leukemia	1,838	7.1	2,360	7.0	7,375	6.8^¶^	11,721	6.5^¶^
Other cancers	5,700	21.5	7,175	20.9^¶^	21,840	19.7^¶^	33,496	18.1^¶^

* Per 100,000 persons, age-adjusted to the 2000 U.S. standard population.

^†^ Counties were identified using the U.S. Department of Agriculture Economic Research Service 2013 vintage rural-urban continuum code, which categorizes nonmetropolitan counties by degree of urbanization and adjacency to a metropolitan area, and metropolitan counties by the population size of their metropolitan area (https://www.ers.usda.gov/data-products/rural-urban-continuum-codes).

^§^ Data from the National Vital Statistics System.

^¶^ Rates were significantly different from rates in nonmetropolitan rural areas. (The 95% confidence interval for the rate ratio excluded one.)

** Mutually exclusive racial/ethnic groups are based on information about race/ethnicity that was collected separately and combined for this report. The white, black, American Indian/Alaska Native, and Asian/Pacific Islander race categories are all non-Hispanic. Rates are not presented for those with unknown ethnicity.

By cancer site, nonmetropolitan rural counties experienced higher death rates than all other counties for cancers of the lung, colon and rectum, prostate, and cervix; for female breast cancer, death rates were higher than in metropolitan counties with <1 million population but similar to rates of other counties (Figure 1). In addition, compared with all other counties, nonmetropolitan rural counties experienced higher death rates from cancers of the oral cavity and pharynx and kidney (Table 2). Death rates for cancers of the esophagus, larynx, and bladder, melanoma, and leukemia in nonmetropolitan rural counties were similar to those in nonmetropolitan urban counties but higher than in metropolitan counties, whereas death rates for cancers of the ovary and brain were higher than in metropolitan counties with ≥1 million population and similar to rates of other counties. Death rates in nonmetropolitan rural counties were lower than all other counties for liver cancer, lower than in metropolitan counties for stomach cancer, and lower than in metropolitan counties with ≥1 million population for uterine cancer, whereas death rates for non-Hodgkin lymphoma were lower than in nonmetropolitan urban counties but higher than in metropolitan counties. Death rates for pancreatic cancer and myeloma were similar in nonmetropolitan rural counties compared with all other counties. 

**FIGURE 3:**
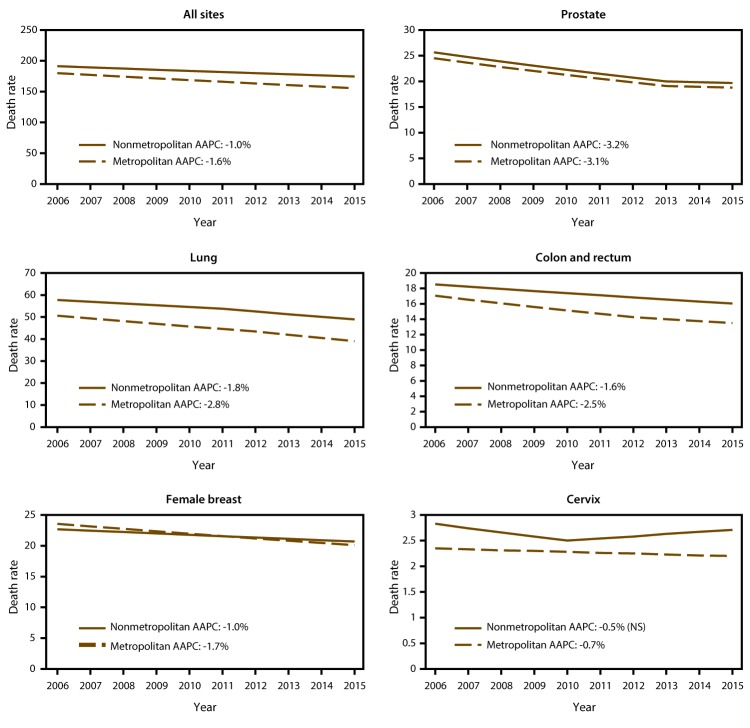
Trends* in annual age-adjusted death rates among persons of all ages for common cancers in nonmetropolitan and metropolitan counties,^†^ by year of death — United States, 2006–2015 **Source:** CDC, National Center for Health Statistics. National Vital Statistics System, mortality data. Atlanta, GA: CDC. **Abbreviations:** AAPC = average annual percentage change; NS = not significant. * Trends were measured with AAPC in annual rates (per 100,000, age-adjusted to the 2000 U.S. standard population) calculated using joinpoint regression, which allowed different slopes for two periods; the year at which slopes changed could vary by county classification. To determine whether AAPC was significantly different from zero, a t-test was used for 0 joinpoints, and a z-test was used for 1 joinpoint. Rates were considered to increase if AAPC >0 (p<0.05) and to decrease if AAPC <0 (p<0.05); otherwise rates were considered stable. All AAPCs were significantly different (p<0.05) from zero unless otherwise indicated (by NS). ^†^ Nonmetropolitan and metropolitan counties were identified using the U. S. Department of Agriculture Economic Research Service 2013 vintage rural-urban continuum code (https://www.ers.usda.gov/data-products/rural-urban-continuum-codes). AAPCs differed significantly between nonmetropolitan and metropolitan counties for cancers of all sites, the lung, the colon and rectum, and the female breast but did not differ for cancers of the prostate or cervix.

During 2006–2015, death rates for all cancer sites combined decreased more slowly in nonmetropolitan counties (-1.0% per year) than in metropolitan counties (-1.6% per year), widening differences in rates (Figure 3). Similarly, differences in death rates also increased for lung and colorectal cancers because rates decreased more slowly in nonmetropolitan counties. Although breast cancer death rates had been lower in nonmetropolitan counties, they decreased more slowly than in metropolitan counties, resulting in similar rates. Cervical cancer death rates were stable in nonmetropolitan counties but decreased slightly in metropolitan counties. Death rates for prostate, stomach, and ovarian cancers; non-Hodgkin lymphoma; and leukemia decreased in nonmetropolitan counties at the same rate as in metropolitan counties but decreased more slowly than in metropolitan counties for laryngeal cancer (supplemental Table 2 https://stacks.cdc.gov/view/cdc/45451). Death rates for melanoma and for cancers of the esophagus and kidney decreased in metropolitan counties but were stable in nonmetropolitan counties (supplemental Table 2 https://stacks.cdc.gov/view/cdc/45451). Death rates for thyroid cancer, oral cavity and pharyngeal cancer, and myeloma were stable both in nonmetropolitan and metropolitan counties (supplemental Table 2 https://stacks.cdc.gov/view/cdc/45451). Death rates increased at about the same rate in nonmetropolitan and metropolitan counties for uterine and brain cancers but increased faster in nonmetropolitan counties for liver cancer (supplemental Table 2 https://stacks.cdc.gov/view/cdc/45451). Pancreatic cancer death rates increased in nonmetropolitan counties but were stable in metropolitan counties, whereas bladder cancer death rates increased in nonmetropolitan counties but decreased in metropolitan counties (supplemental Table 2 https://stacks.cdc.gov/view/cdc/45451).

## Discussion

During the most recent 5-year period with available data, nonmetropolitan rural counties had lower average annual age-adjusted incidence rates (2009–2013) for all cancer sites combined, but higher average annual age-adjusted death rates (2011–2015) for all cancer sites combined, than nonmetropolitan urban and metropolitan counties. During the most recent 10-year period with available data (2004–2013), the annual age-adjusted overall cancer incidence rates decreased equally, by approximately 1% per year, both in nonmetropolitan and metropolitan counties; however, the annual age-adjusted overall cancer death rates (2006–2015) decreased more slowly in nonmetropolitan counties (-1.0% per year) than in metropolitan counties (-1.6% per year), increasing existing differences in rates.

Nonmetropolitan rural counties had higher incidence and death rates for cancers related to smoking (e.g., lung and laryngeal cancers) and cancers that can be prevented by screening (i.e., colorectal and cervical cancers). Rates for these cancers also decreased more slowly in nonmetropolitan counties than in metropolitan counties, increasing disparities. Nonmetropolitan rural counties also had higher incidence rates of cancers related to human papillomavirus (HPV) (cancers of the cervix, oral cavity and pharynx) but lower incidence rates of cancers related to other infectious agents such as liver (which can be caused by hepatitis B virus and hepatitis C virus) and stomach cancer (which can be caused by infection with *Helicobacter pylori* bacteria). Compared with metropolitan counties, nonmetropolitan counties had lower incidence rates for breast and prostate cancer that persisted over time but had similar breast cancer death rates and slightly higher prostate cancer death rates.

### Risk Factor Prevalence

Many cancers are caused by modifiable risk factors, such as cigarette smoking and other tobacco use, secondhand smoke exposure, excess body weight, insufficient physical activity, alcohol use, excessive exposure to ultraviolet rays from the sun and tanning beds, and exposure to cancer-causing infectious agents (*9*). Differences in risk factors such as cigarette smoking could account for some of the disparities presented in this report. Previous research has shown that residents of nonmetropolitan areas, compared with residents of metropolitan areas, have higher rates of cancer risk factors such as cigarette smoking, obesity, and physical inactivity (*4*). Results from the 2013 Behavioral Risk Factor Surveillance System indicated that adults who lived in rural areas, compared with those who lived in nonrural areas, had the lowest prevalence (27%) of reporting at least four of five healthy behaviors defined as current nonsmoking, maintaining normal body weight, nondrinking or moderate drinking, meeting aerobic leisure-time physical activity recommendations, and getting enough sleep (*10*). Many of these healthy behaviors are associated with a reduced risk for cancer (*9*). By risk factor, the study found that adults who lived in rural areas had the lowest prevalence of current nonsmoking (i.e., the highest prevalence of current smoking), maintaining normal body weight (i.e., the highest prevalence of being overweight or obese), and meeting aerobic leisure-time physical activity recommendations (i.e., the highest prevalence of not getting enough physical activity), each of which is associated with increased cancer risk but the highest prevalence of nondrinking or moderate drinking, which could be associated with reduced cancer risk (*10*).

Certain cancer risk factors can be addressed through clinical preventive services. As of 2017, the U.S. Preventive Services Task Force has recommended that all adults be screened for tobacco use and excessive alcohol use and offered counseling and intervention as needed and recommended the use of low-dose aspirin to prevent colorectal cancer and cardiovascular disease among adults who are considered to be at high risk for cardiovascular disease based on specific criteria (https://www.uspreventiveservicestaskforce.org/Page/Name/recommendations). A higher percentage of residents in nonmetropolitan areas than in metropolitan areas was uninsured (*11*), potentially limiting access to preventive services covered by insurance. The Advisory Committee on Immunization Practices recommends vaccination against two cancer-causing viruses, HPV (https://www.cdc.gov/vaccines/hcp/acip-recs/vacc-specific/hpv.html) and hepatitis B virus (https://www.cdc.gov/vaccines/hcp/acip-recs/vacc-specific/hepb.html). Data from the 2014 National Immunization Survey among persons aged 13–17 years indicate that vaccination rates for hepatitis B were similar in nonmetropolitan and metropolitan counties but that the vaccination rates for HPV among both boys and girls were lower in nonmetropolitan counties than in metropolitan counties (*11*).

In addition, disparities could be attributed to differences in adherence to screening guidelines. As of 2017, the U.S. Preventive Services Task Force has recommended population-based screening for colorectal, female breast, and cervical cancers among persons at average risk and for lung cancer in persons at high risk (https://www.uspreventiveservicestaskforce.org/Page/Name/recommendations). Cancer screening is an important aspect of cancer prevention and control because screening can prevent cancer by finding and treating precancerous changes before they progress to cancer, and screening can control cancer by detecting tumors at the earliest stages when they are most treatable. Cervical and colorectal cancer screening tests can detect both precancerous lesions and cancer at an early stage, thus preventing both cancer occurrence and cancer death; breast and lung cancer screening tests can detect cancer at an early stage, thus reducing cancer deaths, although they do not prevent cancer from developing. Data from the 2010 National Health Interview Survey indicated that rates of breast and cervical cancer screening were similar in metropolitan and nonmetropolitan counties but that colorectal cancer screening rates were lower in nonmetropolitan counties (*12*). Increased use of cancer screening might reduce rates of late-stage cancer; data from this report indicate that, compared with metropolitan counties, nonmetropolitan counties generally had higher rates of and slower decreases in late-stage lung cancer and late-stage colorectal cancer but similar rates of late-stage cervical cancer and lower rates of late-stage breast cancer. The data in this report also indicate that nonmetropolitan counties experienced higher death rates from colorectal and cervical cancer. Higher death rates might reflect lack of access to cancer screening services, follow-up to abnormal tests, quality care for cancer patients, and cancer survival care (*4*).

### Evidence-Based Interventions

An important component of cancer prevention and control is implementation of interventions at both the individual level and the population level (*13*). Interventions can be implemented to reduce cancer risk factors, particularly tobacco use, secondhand smoke exposure, alcohol use, and excessive exposure to ultraviolet rays from the sun and tanning beds, and to promote protective factors such as vaccination against cancer-related infectious diseases including HPV and hepatitis B virus, getting enough physical activity, and maintaining a healthy weight. Program and policy interventions proven to increase cancer screening and vaccination rates and to prevent tobacco use, excessive alcohol use, obesity, and physical inactivity have been reviewed and evaluated in *The Guide to Community Preventive Services* (https://www.thecommunityguide.org). The CDC’s National Comprehensive Cancer Control Program brings together diverse partners to plan, implement, and evaluate these interventions in their own communities (https://www.cdc.gov/cancer/ncccp).

CDC also supports the National Breast and Cervical Cancer Early Detection Program, which addresses health disparities among women who are uninsured or underinsured, a common characteristic in rural populations (https://www.cdc.gov/cancer/nbccedp). Although this program has focused on direct screening services, emphasis is shifting toward population-based interventions, similar to those used by the Colorectal Cancer Control Program (https://www.cdc.gov/cancer/crccp). The goal is to work with health care systems, health care payers, health care purchasers, and other partners to implement interventions that address barriers such as providing transportation assistance, having clinics with modified hours, and providing assessment and feedback reports on clinical practice performance. Communities with lower screening rates or higher incidence of and mortality from cancer might particularly benefit from these interventions.

CDC’s National Tobacco Control Program promotes ongoing work to reduce tobacco use through support to state and territorial health departments (https://www.cdc.gov/tobacco/stateandcommunity/tobacco_control_programs/ntcp/index.htm). Compared with urban and metropolitan populations, residents of rural areas have a higher smoking prevalence, start smoking at a younger age, smoke more heavily, use smokeless tobacco at twice the rate, and are more likely to be exposed to secondhand smoke (*14*). Evidence-based strategies to reduce tobacco-related disparities include increasing the number of persons covered by comprehensive smoke-free laws; increasing the price of tobacco products; reducing exposure to targeted tobacco industry advertising, promotions, and sponsorship; and improving the availability and accessibility of cessation services (*14*).

### Healthy People 2020

Many of the *Healthy People 2020* objectives for cancer mortality (all sites, lung, colorectal, cervical, prostate, oral cavity and pharyngeal, and melanoma) were met in metropolitan counties with ≥1 million population; however, only one (prostate cancer) was met in nonmetropolitan rural and urban counties. Regarding objectives for incidence, the objective for colorectal cancer was met in metropolitan counties with <1 million population, and the objective for late-stage breast cancer incidence was met in nonmetropolitan rural and urban counties. Because late-stage incidence rates correspond with overall incidence rates, the lower rate of late-stage breast cancer incidence in nonmetropolitan counties likely reflects a lower baseline breast cancer incidence rather than a difference in late-stage diagnoses. These disparities indicate that not all persons are benefitting equally from initiatives to achieve *Healthy People 2020* objectives and that more work is needed to meet these objectives. Furthermore, using an overall rate, which largely reflects the experience of the heavily populated metropolitan counties, might mask underlying disparities by geographic region such as those highlighted in this report.

## Limitations

The findings in this report are subject to at least five limitations. First, delays in cancer reporting might result in an underestimation of certain cancers; reporting delays are more common for cancers such as melanoma and prostate cancer that are diagnosed and treated in nonhospital settings such as physicians’ offices. Second, incidence was not classifiable by county classification for all states; therefore, these results might not apply to states excluded from the analyses. Third, differences in rates and trends might be statistically, but not clinically, significant. Fourth, analyses of trends should be carefully interpreted; certain rates might be actually increasing or decreasing even though the trend is not statistically significant. Finally, this report used the most recent data available, through 2013 for incidence data and 2015 for mortality data; caution should be used when comparing incidence and death rates because of the difference in years and population coverage.

## Future Directions

This report demonstrates the value of examining cancer incidence and death rates among nonmetropolitan and metropolitan area by cancer type. Examination of county-level patterns within states might be helpful in determining areas with disproportionately high cancer incidence or death rates. Using tools such as State Cancer Profiles (https://statecancerprofiles.cancer.gov) might help identify prevalence of risk factors and use of screening tests that might affect these rates.

## Conclusion

Although nonmetropolitan rural counties had a lower incidence of cancer overall, the incidence of certain cancers, including lung cancer, was higher, as was overall cancer mortality. Many cancers are linked to modifiable risk factors, such as tobacco use, and use of cancer screening tests. Evidence-based strategies to improve health-related behaviors, use of vaccinations that prevent infections with cancer-causing viruses, and use of cancer screening tests, particularly among persons who live in nonmetropolitan rural and urban counties, might help achieve *Healthy People 2020* objectives to reduce cancer morbidity and mortality.
